# Fish Scale Gelatin Nanofibers with *Helichrysum italicum* and Lavandula latifolia Essential Oils for Bioactive Wound-Healing Dressings

**DOI:** 10.3390/pharmaceutics15122692

**Published:** 2023-11-28

**Authors:** Carmen Gaidau, Maria Râpă, Maria Stanca, Mariana-Luiza Tanase, Laura Olariu, Rodica Roxana Constantinescu, Andrada Lazea-Stoyanova, Cosmin-Andrei Alexe, Madalina Tudorache

**Affiliations:** 1 The National Research & Development Institute for Textiles and Leather, Division Leather and Footwear Research Institute, 31251 Bucharest, Romania; carmen.gaidau@icpi.ro (C.G.); rodica.roxana@yahoo.com (R.R.C.); cosminandrei.alexe@icpi.ro (C.-A.A.); 2 Faculty of Materials Science and Engineering, National University of Science and Technology Politehnica Bucharest, 060042 Bucharest, Romania; 3 SC Biotehnos SA, 3-5 Gorunului Street, 075100 Otopeni, Romania; luiza.craciun@biotehnos.com (M.-L.T.); lolariu@biotehnos.com (L.O.); 4 National Institute for Laser, Plasma and Radiation Physics, 409 Atomistilor Street, 077125 Magurele, Romania; andrada@infim.ro; 5 Laboratory for Quality Control and Process Monitoring, Faculty of Chemistry, University of Bucharest, 4-12 Regina Elisabeta Boulevard, 030018 Bucharest, Romania; madalina.sandulescu@g.unibuc.ro

**Keywords:** essential oils, fish scale gelatins, nanofibers, electrospinning, wound healing

## Abstract

Essential oils are valuable alternatives to synthetic antibiotics that have the potential to avoid the pathogen resistance side effects generated by leather. *Helichrysum italicum* and Lavandula latifolia essential oils combined with fish scale gelatin were electrospun using a coaxial technique to design new bioactive materials for skin wound dressings fabrication. Fish scale gelatins were extracted from carp fish scales using two variants of the same method, with and without ethylenediaminetetraacetic acid (EDTA). Both variants showed very good electrospinning properties when dissolved in acetic acid solvent. Fish scale gelatin nanofibers with *Helichrysum italicum* and Lavandula latifolia essential oil emulsions ensured low microbial load (under 100 CFU/g of total number of aerobic microorganisms and total number of yeasts and filamentous fungi) and the absence of *Staphylococcus aureus* ATCC 6538, *Escherichia coli* ATCC 10536, and *Candida albicans* ATCC 1023 as compared to fish scale gelatin without essential oils, which recommends them for pharmaceutical or topical applications. A scratch-test performed on human dermal fibroblasts proved that the biomaterials contributing to the wound healing process included fish scale gelatin nanofibers without EDTA (0.5% and 1%), fish scale gelatin nanofibers without EDTA and *Lavandula latifolia* essential oil emulsion (1%), fish scale gelatin nanofibers with EDTA (0.6%), and fish scale gelatin nanofibers with EDTA with *Helichrysum italicum* essential oil emulsion (1% and 2%).

## 1. Introduction

Electrospun nanofibers are known as excellent materials for medical, energy storage, and environmental engineering applications due to their large surface-to-volume ratio, high porosity with nanoscale interstitial spaces, and interconnectivity [1]. Nanofibers have been fabricated from synthetic or natural polymers [2,3], carbon-based and semiconductor nanomaterials [4], or different composites [5,6,7,8,9]. The nanofiber scaffold is considered one of the most researched biomaterials due to its versatility in mimicking the extracellular matrix (EMC), which is favorable for cell adhesion and proliferation in regenerative medicine and tissue engineering [10].

Wound dressings are important materials in traumatic, thermal, chronic, diabetic, or surgical wound management due to their fluid absorption and microbial protection properties [11,12,13,14].

The main stages of acute wound healing are generally considered: homeostasis, inflammation, proliferation, reepithelialization, and remodeling [14]. The healing process is very complex and involves blood loss control in the homeostasis stage by activation of clotting factors, growth factors activation with inflammatory effect, proliferation of cells responsible for dermis restoration, new tissue formation in the reepithelialization stage, and finally, the regression of capillaries and apoptosis of macrophages and fibroblasts in the remodeling stage. When the healing stages are disturbed in one of the healing phases, the wound becomes chronic and the healing rate is lower and lasts more than 12 weeks [14]. Normal wound healing is a dynamic and complex process that begins at the time of injury and involves all resident cells as well as migratory cell populations, the extracellular matrix, and the action of soluble mediators [15]. The optimal wound healing involves the following events: (1) rapid hemostasis; (2) appropriate inflammation; (3) mesenchymal cell differentiation, proliferation, and migration to the wound site; (4) suitable angiogenesis; (5) prompt reepithelialization (regrowth of epithelial tissue over the wound surface); and (6) proper synthesis, cross-linking, and alignment of collagen to provide strength to the healing tissue [16]. The cascade of initial vasoconstriction of blood vessels and platelet aggregation is designed to stop bleeding. This is followed by an influx of a variety of inflammatory cells, starting with neutrophils. These inflammatory cells, in turn, release a variety of mediators and cytokines to promote angiogenesis, thrombosis, and reepithelialization. Fibroblasts, in turn, establish extracellular components that will serve as support structures. The inflammatory phase is characterized by hemostasis, chemotaxis, and increased vascular permeability, limiting further damage, closing the wound, removing cellular debris and bacteria, and promoting cell migration. The inflammatory stage usually lasts a few days. The proliferative phase is characterized by the formation of granulation tissue, reepithelialization, and neovascularization. This phase can last several weeks. The maturation and remodeling phase is where the wound reaches maximum strength as it matures [17].

The electrospinning technique allows the use of biocompatible, biodegradable polymers with the addition of drugs or bioactive substances, increasing the efficiency of the wound-healing mechanism [18].

Electrospinning is an electrohydrodynamic technique [19] that involves the application of an electric field between the solution ejected from the tip of the needle and the metallic collector. The liquid droplets are electrically charged and stretched in a cone shape when the solvent evaporates, and solid nanofibers deposit on the collector cover. The nanofiber’s characteristics depend on the solution parameters (viscosity, concentration, molecular weight, surface tension, and conductivity), electrospinning parameters (voltage, flow rate, feeding rate, distance between the needle tip and collector), and environmental conditions (temperature and humidity).

The recent innovations in fiber-based scaffolds for engineering skin tissue were listed [20] and included some composites based on collagen, such as polypyrrole/chitosan/collagen [21], polycaprolactone (PCL)/gelatin/MgO/endometrial stem cells [22], collagen–graphene oxide loaded with basic fibroblast growth factors [23], PCL/gelatin/acetylated cellulose nanofibers [24], fish collagen/PCL cross-linked with chitooligosaccharides [25], and ZnO quantum dots with PCL/collagen fibers [26]. The tested nanofibers fabricated by direct or coaxial electrospinning showed increased cell adhesion, growth, and proliferation. They also demonstrated capabilities such as full-thickness skin wound closure in mice, increased healing and skin regeneration in rats, or enhanced vascularization and promotion of wound healing in the early stage of the healing process. Fiber-based scaffolds using collagen extracted from fish scales are not included in recent innovations due to limited research on fiber fabrication with this material.

Collagen from fish scales has attracted research attention due to its complex composition, including collagen, keratin, mucin, hydroxyapatite, chitin [27], calcium carbonate [28], iron, and magnesium [29]. These components can potentially enhance the regenerative characteristics of the wound-healing process. The addition of antimicrobial and antioxidant natural extracts to fish scale collagen represents an innovative approach to designing efficient biomaterials for wound healing, the topic of our research.

Essential oils are concentrated extracts from fruit peels, barks, leaves, entire plants, roots, or flowers [30], with a complex composition of saturated and unsaturated hydrocarbons, alcohol, aldehydes, esters, ethers, ketones, oxides phenols, and terpenes [31]. Their use as complementary remedies is known for numerous diseases like depression, indigestion, headache, insomnia, muscular and joint pain, respiratory problems, and skin and urinary infections, among others [32]. The research has shown that when essential oils are associated with synthetic antibiotics, they can enhance the antibiotics’ efficiency and reduce their use, resulting in fewer side effects [32].

*Helichrysum italicum* essential oil is known for its antibacterial, antifungal, antiviral, and anti-inflammatory properties due to the composition of flavonoids and terpenes (effective against *Staphylococcus aureus*), acetophenones, phloroglucinol, and terpenoids (with antifungal properties against *Candida albicans* and inhibitory activity against pro-inflammatory mediators). The anti-erythematous activity of *Helichrysum italicum* was demonstrated by in vivo tests, and, meanwhile, the low cytotoxicity and genotoxicity were highlighted [33].

*Lavandula angustifolia* was used in alginate/PEO nanofibers and tested for burn wound healing with efficiency due to the antimicrobial and anti-inflammatory mechanisms. The antimicrobial tests of lavender essential oil showed antibacterial efficiency against *Staphylococcus aureus*, and in vivo tests demonstrated the cytokines reductions with an effect on the anti-inflammatory process [34]. The inhibitory effect of lavender essential oil on the nosocomial bacteria formation shows promise as a property for new biomaterials, serving as an alternative to synthetic antimicrobials [35]. Another study has revealed the antibacterial and antifungal properties of *Lavandula angustifolia* and *Helichrysum italicum* essential oils against nine strains of bacteria, as well as their cyto/genotoxic effects on *Allium cepa* cells and human lymphocytes. However, less efficiency was recorded for *Helichrysum italicum* essential oil against Gram-positive bacteria [36].

The lack of information regarding the effect of essential oils on cell toxicity was recently highlighted [37]. A review pointed out that although there are many studies on the antimicrobial and healing effects of essential oils, there is a limited amount of research available regarding their efficacity in promoting human wound healing [38]. Therefore, our research focused on a new approach regarding the fabrication of fish scale gelatin nanofibers with and without essential oils and their skin restructuration properties. The use of only natural biopolymers, eco-friendly solvents, and naturally occurring secondary metabolites, rich in bioactive components, represents an original approach with potential applications for the efficient healing of skin wounds. Our original findings include the improvement of noncytotoxicity concentrations of fish scale nanofibers with essential oil emulsions and an impressive wound healing rate of 149%, with the prospect for the development of new and efficient wound dressings.

## 2. Materials and Methods

### 2.1. Materials

*Helichrysum italicum* (lavender) (L) and Lavandula latifolia (immortelles) (I) essential oils of EOBBD quality (Essential Oil Botanically and Biochemically Defined) were acquired from PURAROM Laboratories SRL (Savinesti, Romania). The Helichrysum italicum essential oil main composition according to GC–MS analyses is composed of 32.45% neryl acetate (Figure S1a), 18.87% α-pinene, 10.46% gamma-curcumene, 2.61% limonene, 4.78% italidione, and 4.15% neryl propionate. The Lavandula latifolia essential oil main composition according to GC–MS analyses is 41.59% linalool (Figure S1b), 27.66% 1,8-cineol, and 11.97% camphor. Tween 20 and Tween 80 were purchased from Redox Research and Analytic SRL (Otopeni, Romania) and used for essential oils emulsification.

Fish scales (F) were collected from a carp fish skin (*Cyprinus carpio*) weighing 2 kg, purchased from the fishery (Carrefour, Bucharest, Romania).

The reagents used in microbiological tests such as tryptic soy broth culture medium (TSB), tryptone soy agar culture medium (TSA), enumeration agar (EA), culture medium of nutrient broth (NB), soybean casein digest lecithin polysorbate 80 medium (SCDLP), and Sabouraud dextrose agar medium (SDA) were of analytical grade (Mediclim, Otopeni, Romania). The microorganism strains of *Staphylococcus aureus* ATCC 6538, *Escherichia coli* ATCC 10536, and *Candida albicans* ATCC 10231 were supplied by Mediclim (Otopeni, Romania). The other chemicals used were of analytical grade.

Cytotoxicity and the “scratch-test” were performed with the following materials: human dermal fibroblast (normal cell line HS27—ATCC^®^ CRL-1634™), DMEM (Dulbecco’s Modified Eagle’s medium/nutrient mixture F-12 Ham (Sigma-Aldrich, St. Louis, MO, USA), fetal bovine serum (Sigma-Aldrich), antibiotic antimycotic solution (100×) (Sigma Aldrich), Trypsin-EDTA 1x (Sigma Aldrich), phosphate buffer (PBS) (Sigma Aldrich), trypan blue stain 0.4%, CellTiter 96^®^ Aqueous One Solution Cell Proliferation Assay (Promega, Madison, WI, USA), and CytoTox 96^®^ Non-Radioactive Cytotoxicity Assay from Promega.

### 2.2. Methods

#### 2.2.1. Fish Scale Gelatin Preparation

Carp fish scales’ characteristics were found to be as follows: dry matter of 38.33%, total ash of 8.5%, total nitrogen of 5.08, protein content of 30.48%, extractible substances with organic solvent of 0.17%, and pH of 7.23.

**Figure 1:**
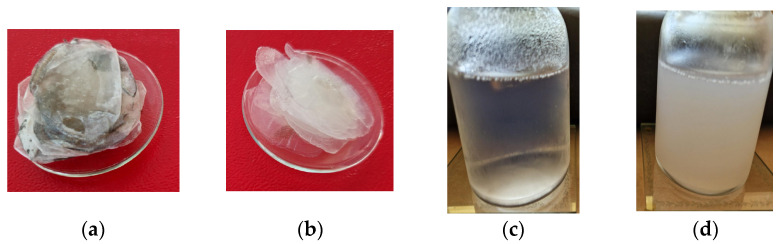
Images for: (**a**) Raw carp fish scales; (**b**) Washed and degreased fish scales; (**c**) Gelatins with EDTA; and (**d**) Gelatins without EDTA.

Fish scale gelatin was prepared from carp fish scales (Figure 1a) following an adapted method from the literature [39], according to which the fish scales were washed with 5% *w*/*v* NaCl and 4% *w*/*v* NaOH to release soluble proteins and mucins and degreased with n-butanol (Figure 1b). In the first variant, the fish scales were treated for 16 h with ethylene diamine tetraacetic acid (EDTA) and for 3 h with acetic acid 99% *v*/*v* with the aim to solubilize the minerals (Figure 1c). In the second variant, the fish scales were not treated with EDTA. Instead, they were treated only with acetic acid 99% *v*/*v* for 3 h. Finally, the fish scale gelatins were extracted from chopped fish scales by heating at 60 °C for 12 h in distilled water (Figure 1d). Solid gelatins were prepared by drying fish scale gelatin at 60 °C.

The gelatin extraction yield was calculated according to the formula [40,41]:

(1) $$\%\;Yield=\frac{Weight\;of\;dried\;gelatin}{Weight\;of\;dried\;scales}\times 100\;$$

#### 2.2.2. Essential Oil Emulsions Preparation

Each emulsion contains essential oil dissolved in ethanol at a ratio of 1:1, Tween 20, Tween 80, and distilled water. The essential oil:surfactant ratio was 4:1, and the amount of water from the total volume was 82%. Tween 80 and Tween 20 were used as non-ionic surfactants in this study due to their high solubility in essential oil. The mixture underwent continuous stirring at 800 rpm for 30 min, until it formed a stable, translucent, and fine oil-in-water (O/W) emulsion (Figure S2).

The advantage of each emulsion preparation consists in preserving the antimicrobial activity of essential oils due to the environmental stress factors, which led to the incorporation of small amounts of antimicrobial agents.

#### 2.2.3. Preparation of Fish Scale Gelatin Nanofibers without and with Essential Oil Emulsions

The nanofibers were achieved using a TL-Pro-BM Electrospinning machine (Tong Li Tech Co., Ltd., Bao An, Shenzhen, China) consisting of a syringe pump, a high-voltage power supplier, and a grounded conductive drum collector (Figure S3), which was covered with polypropylene sheet of medical grade. An amount of 12.5% (*g*/*v*) of fish gelatin was dissolved in an aqueous solution of acetic acid (9:1 *v*/*v*) by continuous mechanical stirring at 600 rpm for 30 min. The essential oil emulsions were introduced into fish scale gelatin by coaxial electrospinning technique. This process enables the controlled release of bioactive compounds.

The prepared fish scale gelatin nanofibers and fish scale gelatin nanofibers with essential oils are shown in Table 1.

The optimum electrospinning conditions for the processing of fish scale gelatin nanofibers at 26–26.5 °C and relative humidity of 10% are presented in Table 2.

### 2.3. Investigation Methods

#### 2.3.1. Characterization of Fish Scales and Fish Scale Gelatins

The fish scales and fish scale gelatins were analyzed for volatile matters [42], ash content [43], total nitrogen and protein content [44], conductivity [45], pH [46], viscosity (DV2T™ Viscometer Brookfield) at 60 °C [47], and texture (TEX’AN TOUCH 50 N texture analyzer, LAMY RHEOLOGY (Le Carré D’Argent, France). The molecular weight was analyzed by GPC and SDS–PAGE gel electrophoresis (Mini PROTEAN 3 Cell Bio-Rad, Hercules, CA, USA). The proteins were separated in a 12.5% gel run at 30 V for 30 min and at 100 V for 120 min. A molecular weight marker ranging from 10 to 250 kDa (Bio-Rad) was used. The gels were stained with 0.2% Coomassie Brilliant Blue R-250 solution. The lanes and bands ratio were measured with the Gel Doc EZ Imaging System and analyzed with ImageLab software, version no. 6.0.1. The molecular weight determined by GPC was done using an Agilent Technologies instrument (1260 model) (Agilent Technologies, Santa Clara, CA, USA) equipped with a PL aqua gel-OH MIXED-H column (7.5 × 300 mm, 8 µm) and multidetection unit. The flow rate of the mobile phase containing 1 mL min^−1^, the injection volume of the sample was 100 µL, with a temperature of 35 °C for the detectors and column. The calculation of the MW was performed with the Agilent GPC/SEC Software (Version 1.1, Agilent Technologies, Santa Clara, CA, USA).

The analyses were performed In triplicate, and the results were expressed as the average values.

#### 2.3.2. Scanning Electron Microscopy with Energy Dispersive X-ray Spectroscopy (SEM–EDX) Analysis

SEM images for fish scale gelatin nanofibers without essential oil emulsions were analyzed using an FEI Inspect S50 Scanning Electron Microscope equipped with an EDX unit for elemental analysis. All samples were covered with a thin gold layer of about 20 nm to avoid the charging effect by using a sputtering Cressington 108 auto sputter coater device, equipped with a Cressington mtm 20 thickness controller. The secondary electron images were obtained at a 10 mm distance, using 10 kV acceleration voltage and magnification from 50× up to 10,000×. The nanofibers’ average thickness was reported as the mean diameter of a minimum of 50 nanofibers, using Origin’s built-in Gaussian fitting curve software, and the thickness measurement was processed using ImageJ software, version no. 1. 54d.

#### 2.3.3. Antimicrobial Activity

##### Antimicrobial Activity and Minimum Inhibitory Concentrations of Essential Oils

The testing of antimicrobial activity for the lavender and immortelle essential oils was carried out by the disc diffusion method for Gram-positive bacteria (*Staphylococcus aureus* ATCC 6538), Gram-negative bacteria (*Escherichia coli* ATCC 10536), and a yeast (*Candida albicans* ATCC 1023). The bacterial cultures were maintained in tryptic soy broth medium (TSB), and the yeast was cultured in Sabouraud dextrose agar slants (SDA). Stock solutions of the test substances were prepared in dimethyl sulfoxide (DMSO) and kept at room temperature in the dark. Briefly, aliquots of 100 μL of all microbial inoculums in an exponential growth phase with the cell concentration adjusted to 10^5^–10^6^ CFU mL^−1^ were spread over the plate surfaces of TSB for bacteria and SDA for the yeast to ensure sterility. Then, sterile test discs (Φ = 6 mm) were placed into each agar plate.

After the application of essential oil (10 µL/disc), Petri dishes were incubated at 35 ± 2 °C for 16–18 h for the bacteria, and 24 h for *C. albicans*. The antimicrobial effect was evaluated based on the diameter (mm) of inhibition zones. All tests were performed in triplicate, and the mean value ± standard deviation (SD) was taken for further analysis. As a positive control, the standard antibiotic Ampicillin (10 mg) was used, while DMSO served as a negative control.

The quantitative evaluation of antibacterial activities was performed using the microdilution broth method. Each well of the 96-well microtiter plate was aliquoted with 100 µL of Mueller–Hinton broth (MHB). One hundred microliters of MHB were introduced to the 12th well (sterility control), whereas MHB was added to the 11th well (growth control). A volume of 100 µL of each essential oil dissolved in DMSO was added to the first well, and a serial twofold dilution was performed by transferring 100 µL of the suspension to the subsequent wells up to the 10th well; the final 100 µL of the suspension was discarded. A bacterial suspension of 1.5 × 10^8^ CFU/mL was prepared starting from 18 to 24 h solid cultures obtained on TSA and diluted, to achieve a final inoculum density of 1 × 10^7^ CFU. After incubation of the inoculated 96-well plates at 37 °C for 18–24 h, the minimum inhibitory concentration (MIC) values were determined using a conventional plating method. The lowest concentrations of material tested which visibly inhibited growth and respectively determined 99.9% growth inhibition upon subculturing on Mueller–Hinton agar (MHA) plates after overnight incubation at 37 °C were used as the MIC. The results are expressed as the mean ± SD (n = 3).

##### Determination of Microbial Contamination of Fish Scale Gelatin Nanofibers with and without Essential Oil Emulsions

Microbial contamination control seeks to determine the total number of aerobic microorganisms (TAMC) or the absence of pathogenic or conditioned pathogenic microorganisms (TYMC). The conditioned pathogenic microorganisms were *Staphylococcus aureus* TCC 6538, *Escherichia coli* ATCC10536, and *Candida albicans* ATCC1023. The method consists of determining the reduction of the initial concentration of microorganisms inoculum after interaction with fish gelatin nanofibers. The microorganisms were taken from the preserved stocks, and the initial concentrations of unity cells were determined by decimal dilutions. Then, 100 µL of the last dilution of each strain was spread into nutrient agar. A plate with EA was striped, incubated at 37 ± 2 °C for 24 h to 48 h, and then 20 mL of TSB was placed into a 100 mL Erlenmeyer flask. After 24 h of incubation, the plate counts were performed, and the results were kept as a reference for cell growth in the control and test samples. The plates with cell densities similar to that of dilution 10^5^ were considered to have similar CFU values (1.2 × 10^5^ CFU/g for *Staphylococcus aureus*, 1.0 × 10^5^ CFU/g for *Escherichia coli*, and 2.5 × 10^4^ CFU/g for *Candida albicans*). Next, 1.0 ± 0.1 mL of the inoculum was pipetted onto several points over each test sample, and then the sample was placed in vials, then the vials were shaken and 20 mL of SCDLP medium was added immediately. The vials with the test material were kept in an incubator at 37 ± 1 °C for 18–24 h, then 1 mL of the inoculum was taken from the bacterial suspension in the sample, placed in a test tube incorporating 9.0 ± 0.1 mL of NB, and shaken well. An amount of 1 mL of this solution was added to a different test tube containing 9.0 ± 0.1 mL of medium and shaken well. The operations were repeated 10 times so that a series of dilutions were prepared. Afterward, 1 mL of each dilution was pipetted successively into two Petri dishes, while 15 mL of TSA was heated to 45 ± 1 °C in a water bath and added to the Petri dishes. After incubation, the number of colony-forming units on the Petri dishes of the dilution series, on which 30 CFU/g to 300 CFU/g had appeared, were counted.

The results were reported as the total number of bacteria (TAMC), which represents the average CFUs determined on the agar medium with casein and soy hydrolysate and the total number of yeasts and filamentous fungi (TYMC), which represents the average CFUs determined on the Sabouraud agar medium with chloramphenicol.

#### 2.3.4. Cytotoxicity Evaluation for Fish Scale Gelatin Nanofibers with and without Essential Oil Emulsions

To establish the dose–effect relation of the fish scale gelatin nanofibers, as well as the cytocompatibility conditions, a dermal fibroblast standardized cell line (HS27—ATCC^®^ CRL-1634™) was used. Cells were cultured in DMEM (Dulbecco’s Modified Eagle’s medium/nutrient mixture F-12 Ham), supplemented with 10% fetal bovine serum (FBS), 1% Antibiotic Antimycotic Solution (100×), and incubated at 37 °C in a humidified atmosphere with 5% CO_2_. The medium was routinely changed every 3rd day, and at the confluence, cells were subcultured by trypsinization (0.5% trypsin/0.02% EDTA). In all experiments, cells were used between P32 and P37 passage cultures. For testing in the cellular system, the fish scale gelatin nanofibers, with and without essential oil emulsions, were cut in the form of a square with a side of 1.5 cm (F-EDTA; F-EDTA+L; F+EDTA; F+EDTA+L) and 2.5 cm, respectively (F-EDTA+I and F+EDTA+I), sterilized by UV irradiation (30 min, 254 nm), then immersed in 5 mL of complete medium, incubation for 6 h at 37 °C, with shaking. The dilutions were made in a complete medium.

The evaluation of gelatin nanofibers’ cytotoxic effect was performed by establishing the correlation between the decrease in cell viability (MTS test) and the increase in enzyme activity in the culture medium (LDH test). The cells were distributed in 96-well plates with a density of 7000 cells/well and left to adhere for 24 h. They were treated for 48 h with the substance to be tested, according to the work protocol for the kit of specific reagents (MTS/LDH). For the LDH assay, at the end of the incubation period, 25 µL of the supernatant from each well was removed and transferred to another 96-well plate with a flat bottom, and 25 µL was added to the reconstituted substrate to each well, incubated in the dark and at room temperature for 30 min. Then, 25 µL of Stop Solution from the kit was added to each well, and the absorbance at 490 nm was read within a maximum of 1 h after stopping the reaction. In the same experimental model as the LDH test, for MTS dosing, the following procedure was used: to the 65 µL of supernatant remaining in the plate where the adhered cells are, 10 µL of MTS was added, and the plate was incubated for 2 h at 37 °C, in the incubator, forming a colored compound brow. The absorbance at 490 nm was recorded, and the same data processing as for the LDH assay was applied.

To calculate the results and obtain the cytotoxicity curves, the sample/control ratio was calculated and represented graphically according to the concentrations of the tested sample. Thus, by correlating the MTS test with the LDH release test, the effect of the tested product on cell viability can be correctly quantified [48].

#### 2.3.5. The “Scratch-Test” on Human Dermal Fibroblasts

To assess the regenerative potential of the fish scale gelatin nanofibers with and without essential oil emulsions, a wound healing assay was carried out in HS27 cells following a protocol that involves creating a “scratch” in the cell monolayer, capturing images at the beginning and at regular intervals during cell migration to close the scratch, and comparing the images to determine the speed of cell migration. The gelatin nanofibers were evaluated at two different concentrations selected according to the results obtained from the cytotoxicity test. The in vitro wound healing test is a simple and economical method of studying cell migration in vitro. This method is based on the observation that, upon creation of an artificial wound, on a monolayer of confluent cells, cells at the edge of the newly created wound will move until cell–cell contacts are reestablished. The basic steps involve creating a “scratch” in the cell monolayer, capturing images at the beginning and at regular intervals during cell migration to close the scratch, and comparing the images to determine the speed of cell migration. One of the major advantages of this simple method is that it somewhat mimics cell migration in vivo. For example, removing part of the endothelium from blood vessels will induce the migration of endothelial cells (ECs) into the denuded area to close the wound. Another advantage of in vitro tissue is its special suitability to study cell migration through its interaction with the extracellular matrix (ECM) and cell–cell interactions.

The cells are cultured for adherence at a cell density of 60,000–80,000 cells/well, in 24-well plates with untreated flat bottoms, for 24–48 h until they reach confluence. In the cellular monolayer, the artificial wound is created with the automatic device for Autoscratch from Biotek, then the detached cells are removed with PBS, and the samples to be analyzed are introduced into the cellular system. The plates are transferred to the Cytation 5 Imaging reader (BioTek, Agilent, Santa Clara, US) multimodal cell imaging reader for monitoring and acquisition of images for 24 h, at 37 °C/5% CO_2_.

Using image-based cellular analysis, it was possible to accurately quantify the area occupied by cells in the image using a primary mask (Sum Area). The Object Sum Area values were used to generate two additional matrices, namely, wound confluence and maximum wound healing rate. Then, using the appropriate formulas, the wound confluence or the percentage of the initial wound area covered by migrating cells over time and the wound healing rate (μm^2^/h) were calculated. Each parameter was automatically calculated by the wound healing protocol in Gen5 software, version 3.11).

#### 2.3.6. Statistical Analysis

Statistical analysis was performed using Origin 2022. The results were expressed as the average of at least three measurements with the standard deviation values. The one-way ANOVA was used to analyze the differences among the means of the samples compared with the control. We investigated if a single independent variable, or factor, has a measurable effect on the composition of samples. Through statistical analysis, a significant difference between the group’s means is achieved at a level of confidence set at *p* < 0.05.

## 3. Results and Discussions

The fish scale gelatin extraction was performed with high yield values of 33.41% ± 0.40% for fish scale gelatin extracted with EDTA treatment and 25.06% ± 0.32% for fish scale gelatin extracted without EDTA treatment. The yields of acidic extracted fish scale gelatins collected from Coregonus peled, Carp, and Bighead carp fish were reported as 14.92% ± 0.68%, 28.05% ± 0.47%, and 24.17% ± 0.96%, respectively [40]. Other research stated that the gelatin yield extracted from fish skins and fish scales is between 6 and 12%, lower than the mammalian gelatin extraction yield [41]. The improvement of fish gelatin extraction yield is important in order to increase industrial production which is estimated at 1.2% of the gelatin world production [47].

### 3.1. Physical–Chemical Characteristics of Fish Scale Gelatins

The characteristics of two kinds of fish scale gelatins are presented in Table 3 and show the characteristics’ values with differences for the gelatin extracted without EDTA, lower concentration, viscosity, and conductivity with effects on lower gelatin strength and higher pH value. If the salts of scales were removed from the gelatin treated with EDTA, traces of salts can be seen in the case of fish scale gelatin without EDTA treatment.

The influence of the extraction method, with or without EDTA, on molecular weight distribution was analyzed by GPC (Table 3 and Figure S4) and showed that the gelatin with EDTA has higher molecular weight as compared to gelatin without EDTA, in agreement with SDS–PAGE determinations (Figure S5 and Table 4), where a higher concentration of high molecular weight components was recorded. The association of gelatin components can explain the high values for the molecular weight analyzed by GPC (Table 3), reported also by other researchers [49,50].

The presence of higher concentrations of α chains in gelatin (Table 4) influences the gelatin strength [51], confirming the higher Bloom test value recorded for fish scale gelatin with EDTA (Table 3). The high molecular weight of fish scale gelatin can explain the good behavior at electrospinning in acetic acid as an ecological solvent.

### 3.2. Scanning Electron Microscopy–Energy Dispersive-X ray (SEM–EDX)

**Figure 2:**
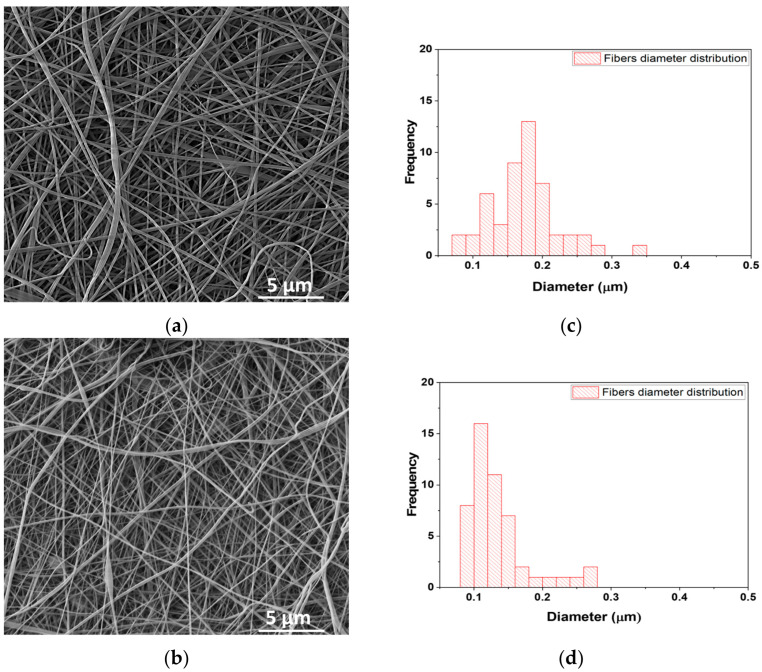
SEM images of fish scale gelatin nanofibers: (**a**) without EDTA, (**b**) with EDTA; Diameter size distribution of nanofibers (**c**) without EDTA, and (**d**) with EDTA.

Analysis of SEM images for fish scale gelatin nanofibers with and without EDTA is shown in Figure 2a,c). The average diameters of fish scale gelatin nanofibers were influenced by gelatin properties. The gelatin with EDTA was electrospun in finer fibers (110.6 nm) (Figure 2d) as compared to the gelatin without EDTA, which generated thicker nanofibers (176.9 nm ± 2.88 nm) (Figure 2b). Fish scale gelatin nanofibers prepared from marine fish scales by using ultrasound showed lower values for the average diameter sizes of 48.11 ± 11.38 nm and 98.75 ± 35.94 nm, probably due to the extraction method. The same nanofibers cross-linked using UV light at 254 nm presented a higher average diameter size of 166.01 ± 53.79 nm and 150.20 ± 64.42 nm, confirming the influence of gelatin characteristics on nanofibers’ morphology [51].

**Figure 3:**
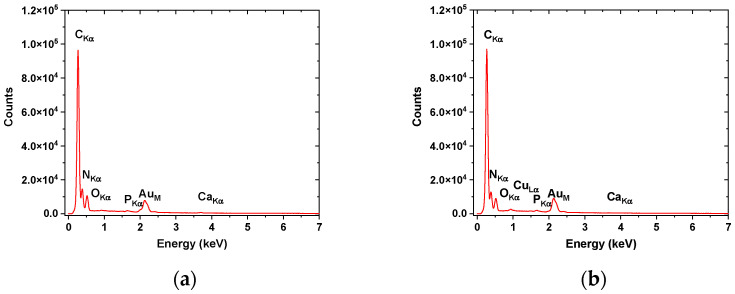
EDX patterns for nanofibers based on fish scales: (**a**) without EDTA and (**b**) with EDTA.

EDX analyses (Figure 3, Table 5) confirmed the higher concentration of Ca and P in nanofibers fabricated from fish scale gelatin without the use of EDTA. The carbon concentration of the F+EDTA sample was found to be higher, probably due to the presence of EDTA traces; meanwhile, complex compounds with Ca and P can contribute to increased concentrations of O and N in the F-EDTA sample. The presence of an Au peak within the EDX graphs (Figure 3) is due to sample preparation covered with an Au layer needed for SEM investigations and is not included in Table 5.

### 3.3. Antimicrobial Properties of Essential Oils

The results of the antimicrobial properties of essential oils are shown in Table 6; Table 7, respectively.

The antimicrobial activity and the MICs of *Helichrysum italicum* and Lavandula latifolia essential oils showed values very close to Ampicillin and Gentamicin. The MIC of lavender essential oil (*L. angustifolia* Mill.) against *S. aureus* ATCC 43300 was found to be between 13.72 ± 0.00 and 18.29 ± 7.92 mg/mL [52]. MICs of *Helichrysum italicum* were found to have the value of 0.312 mg/mL and 0.625 mg/mL against respiratory bacteria, *Haemophilus influenzae*, *H. parainfluenzae*, *P. aeruginosa*, and *S. pneumoniae*. The same study showed that the antimicrobial mechanism is based on the antibiofilm activity *Helichrysum italicum* and cell membrane damage under its activity [53]. From Table 6 and Table 7, it can be seen that the efficiency of essential oils is higher against Gram-positive bacteria as compared to Gram-negative bacteria due to the different structures of outer bacteria cell membranes, as the other research reports also found [54,55].

The mechanism of antimicrobial activity of essential oils is attributed to the ability to change the permeability of cell membranes, protein denaturation, and the inhibition of mitochondrial activities [51]. The main hydrophobic components of *Lavandula latifolia* essential oils (linalool, 1,8-cineol, and camphor) can penetrate the lipophilic layer of the Gram-positive bacteria cell membrane and produce its leakage [56].

The reach composition of *Helichrysum italicum* in neryl acetate, α-pinene, and gamma-curcumene and the synergy with other minor components can explain the antibacterial and antifungal activity [52]. The antimicrobial activity against *S. aureus* was attributed by other authors to its composition in flavonoids and terpenes. Meanwhile, the antifungal properties against *Candida albicans* were attributed to acetophenones, phloriglucinols, and terpenoids components [30]. The increase in cytotoxicity concentrations in the case of the combination of fish scale gelatin with essential oils confirms the favorable influence on cell viability. Lavander essential oil showed a stimulative effect on fibroblast activity in collagen synthesis and increased expression of TGF-β in the wound healing process [57]. Immortelle essential oils presented anti-inflammatory and anti-hematoma activity due to the italidiones’ properties of transiting from the dionic form to the enolic form [58].

### 3.4. Antimicrobial Properties of Fish Scale Gelatin Nanofibers

The influence of essential oils emulsions on the microbial load of fabricated nanofibers is obvious from the results presented in Table 8 and Table 9. The values found for TAMC, TYMC (under 100 CFU), and the absence of *S. aureus*, *E. coli*, and *C. albicans* allowed us to state that the analyzed fish scale gelatin nanofibers with essential oil emulsions are suitable for topical or pharmaceutical products, according to the European Pharmacopoeia.

### 3.5. Evaluation of the Cytotoxic Effect of Fish Scale Gelatin Nanofibers and Helichrysum Italicum and Lavandula Latifolia Essential Oils with Fish Scale Gelatin Nanofibers on Human Dermal Fibroblast Cells

**Figure 4:**
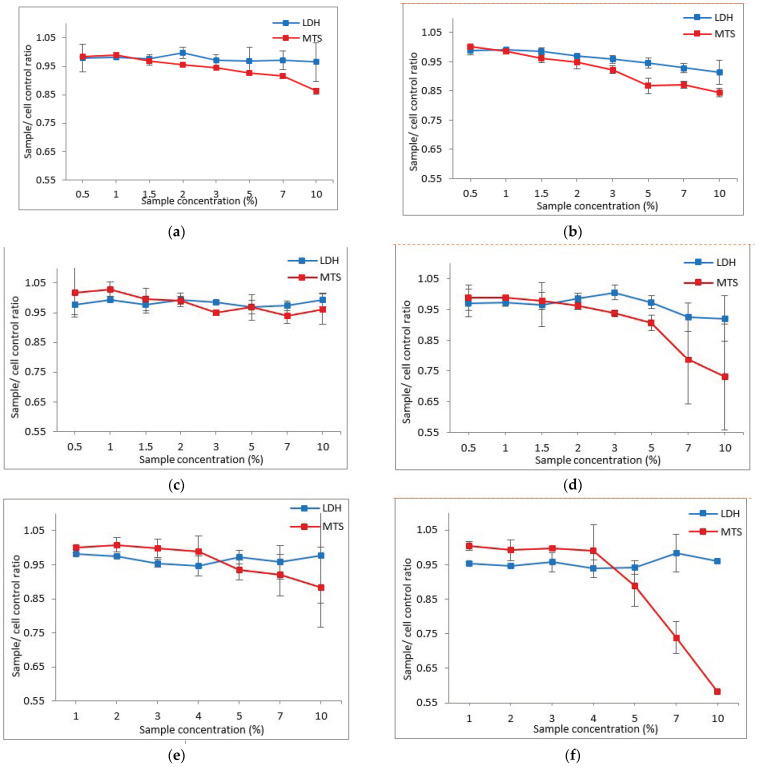
Dose-dependent effect of fish scale gelatin nanofibers on the HS27 cell line viability: (**a**) F-EDTA; (**b**) F+EDTA; (**c**) F-EDTA+L; (**d**) F+EDTA+L; (**e**) F-EDTA+I; (**f**) F+EDTA+I nanofibers. Data are representative of three experiments and are expressed as the ratio of the sample to untreated control; all numerical values are expressed as mean (n = 3) ± standard deviation (SD); *p* > 0.05.

The effect of gelatin nanofibers from fish scales on the viability of human dermal fibroblasts (MTS test) and, respectively, their cytotoxicity (LDH test), was plotted as the ratio between the effect of sample concentration and the cell control (Cc) and is presented in Figure 4. The intersection point between the curves of the two analyzed parameters (LDH and MTS) represents the compound dose at which the signal corresponding to the amount of LDH released into the environment exceeds the signal corresponding to the amount of MTS transformed into formazan, being equivalent to the dose at which cell viability is significantly affected [59,60,61].

Following the analysis of the cytotoxic profile, it is observed that in the case of treatment with gelatin nanofibers from fish scales with EDTA (F+EDTA) and without EDTA (F-EDTA), cell viability is affected (decrease in MTS reduction, simultaneously with increased release cytosolic enzymes LDH) starting with the 1% dose (Figure 4a,b). In the case of exposure of the cells to the different concentrations of F-EDTA+L, the damage to the integrity of the cell membrane, and implicitly to the viability of the cells (by increasing the enzymatic activity in the culture medium and decreasing the MTS reduction), is observed starting with the dose of 2% of the tested product (Figure 4c). In the case of fibroblasts treated with gelatinous nanofibers from fish scales with EDTA with lavender essential oil emulsion (F+EDTA+L), starting with the 1.5% dose, an alteration of cellular metabolism is observed by increasing the amount of LDH released in the extracellular environment (Figure 4d), which indicates an impairment of the integrity of the cell membrane and implicitly of cell viability. In the case of treatment with EDTA-free fish scale gelatin nanofibers and immortelle essential oil emulsion (F-EDTA+I) and EDTA fish scale gelatin nanofibers and immortelle essential oil emulsion (F+EDTA+I), the cells are affected starting with the 4% dose (Figure 4e,f), representing the maximum toxicity dose of the tested compound. The cytotoxicity evaluation of keratin hydrolysates showed values of 1.8% [62], similar to values found for fish scale gelatin nanofibers. The increase in cytotoxicity concentrations in the case of the combination of fish scale gelatin with essential oils confirms the favorable influence on cell viability. Lavander essential oil showed a stimulative effect on fibroblast activity in collagen synthesis and increased expression of TGF-β in the wound healing process [57]. Immortelle essential oils presented anti-inflammatory and anti-hematoma activity due to the italidiones’ properties of transiting from the dionic form to the enolic form [58].

Table 10 summarizes the maximum toxicity dose of the six samples of fish scale gelatin nanofibers containing essential oil emulsions.

Our results are in good agreement with the good cytocompatibility reported for other samples containing gelatin. For instance, Sardareh et al. [63] reported that the incorporation of toxic silver nanoparticles in the PLA/gelatin nanofibers designed for wound dressing application led to inducing a lower level of toxicity on L929 cells. No notable cytotoxic effects on human normal cells were reported when electrospun multilayered mats composed of polycaprolactone/gelatin/polycaprolactone [64], chitosan (CS)/gelatin (GL) and graphene nanosheet (GNS)-CS/GL nanofibers [65], and gelatin/PVA nanofibers [66] were investigated, demonstrating cell proliferation without any signs of necrosis. The good cytotoxicity of a fish gelatin nanofibrous scaffold evaluated based on a cell proliferation study by culturing human dermal fibroblasts (HDFs) was explained due to the hydrophilic surface of nanofibrous fish gelatin, which provides an appropriate environment for the attaching of fibroblast cells [67].

### 3.6. The “Scratch-Test” on Human Dermal Fibroblasts

To evaluate the wound healing process in vitro, the experimental model applied to the human dermal fibroblast cell line (HS27), after a period of 24–48 h for adhesion and cell monolayer formation, includes two series, as follows: a series of unstimulated cells, maintained in culture in the presence of the test substances for 24 h, and a series of cells maintained in culture in the presence of the test substances, simultaneously with the mimicking of acute inflammation: (Tumor necrosis factor (TNF-α), which is an inflammatory cytokine produced by macrophages/monocytes during the acute inflammation phase and is quickly released and initiates inflammation at wound tissues [68]), associated with pro-oxidative conditions (Phorbol myristate acetate (PMA) in an oxidant, which activates neutrophils—the first circulating inflammatory cell to move to the site of the wound, thus being a promoter of acute inflammation [69]), for 24 h. The positive control used in this experimental model is vitamin C, which is known for its role in wound healing in the early stages by reducing inflammation and improving the wound healing process by promoting collagen synthesis and the migration of epithelial cells in the stage of reepithelialization. The concentration of vitamin C was chosen on the basis of published data [70,71] and preliminary tests performed on this specific cell line (unpublished data).

**Figure 5:**
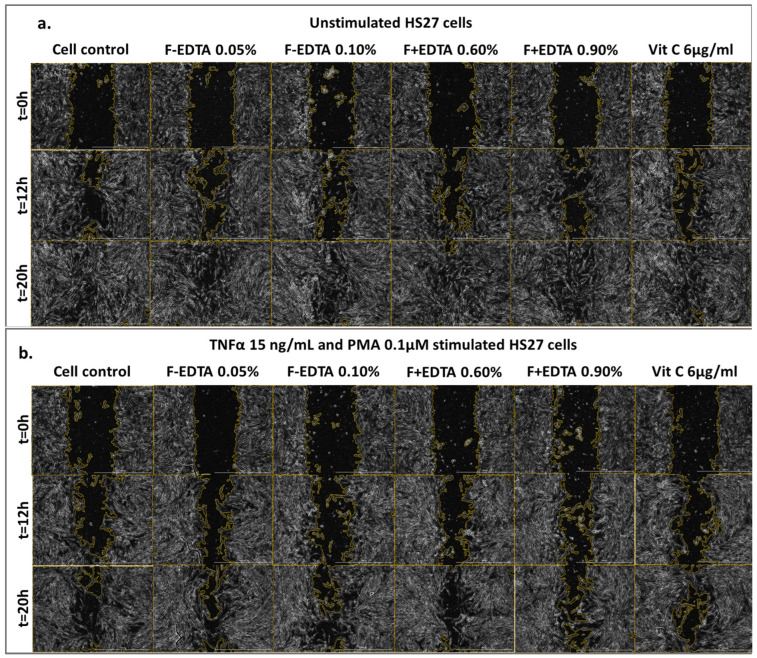
Image-based monitoring HS27 cells migration. Imaging was performed over 20 h using phase contrast and 4x magnification (Scale bar = 1000 µm). Kinetic images shown are for cells treated with fish scale gelatin nanofibers without and with EDTA (F-EDTA and F+EDTA) on the standardized cell line of human fibroblasts–HS27: treated under normal conditions (**a**), and pro-inflammatory stimulated (TNFα 15 ng/mL and PMA 0.1 μM) (**b**).

**Figure 6:**
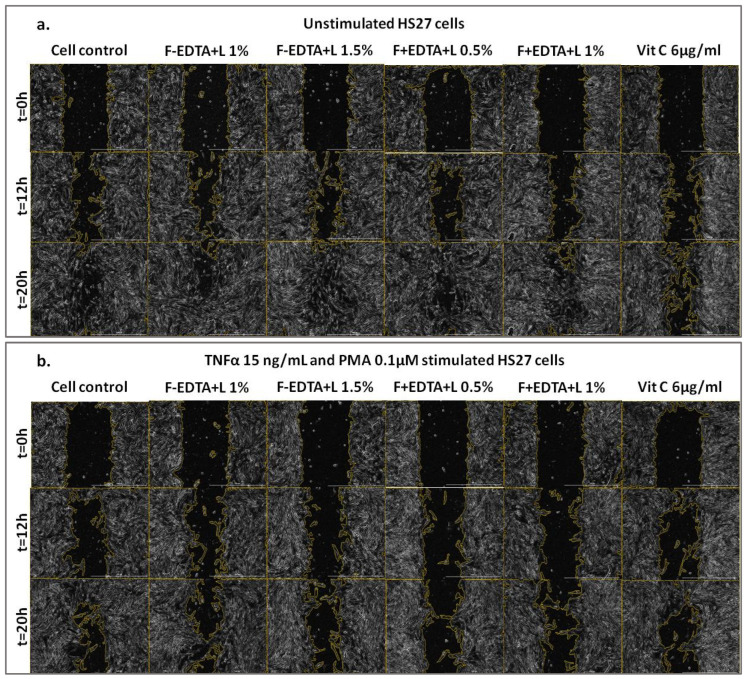
The effect of fish scale gelatin without and with EDTA, containing lavender essential oil emulsion (F-EDTA+L and F+EDTA+L) on the standardized cell line of human fibroblasts–HS27: treated under normal conditions (**a**), and pro-inflammatory stimulated (TNFα 15 ng/mL and PMA 0.1 μM) (**b**). Images were acquired microscopically at different time intervals: t = 0 h, t = 12 h, t = 20 h.

**Figure 7:**
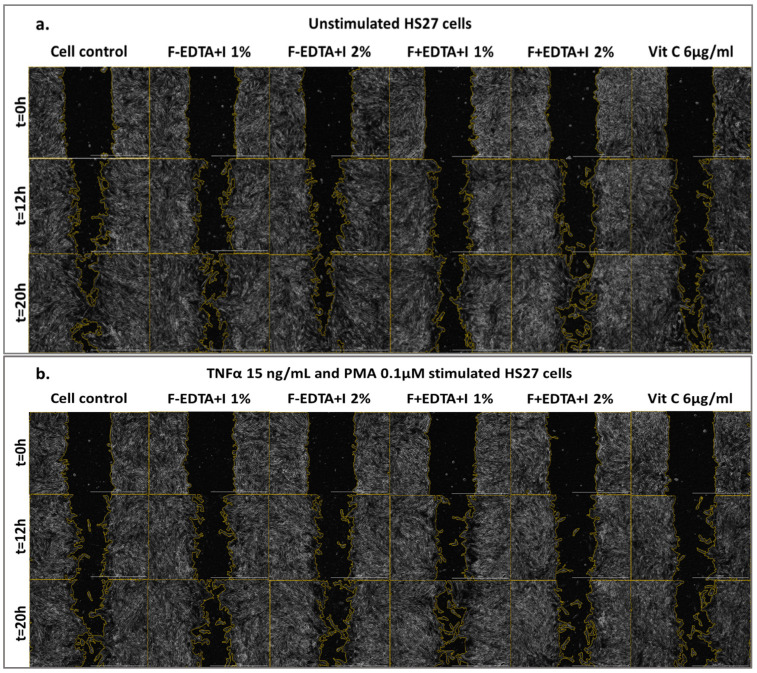
Image-based monitoring HS27 cells migration. Imaging was performed over 20 h using phase contrast and 4x magnification (Scale bar = 1000 µm). Kinetic images shown are for cells treated with fish scale gelatin without and with EDTA containing immortelle essential oil emulsion (F-EDTA+I and F+EDTA+I) on the standardized cell line of human fibroblasts–HS27: treated under normal conditions (**a**), and pro-inflammatory stimulated (TNFα 15 ng/mL and PMA 0.1 μM) (**b**).

Figure 5, Figure 6 and Figure 7 show the images acquired with the automatic microscope, selecting three times from the total monitoring interval of the wound healing kinetics: t = 0 h, 12 h, and 20 h.

For the evaluation of the wound healing process, for each sample of fish scale gelatin nanofibers, two concentrations were selected so that they were lower than or equal to the maximum dose determined in the cytotoxicity assays.

**Figure 8:**
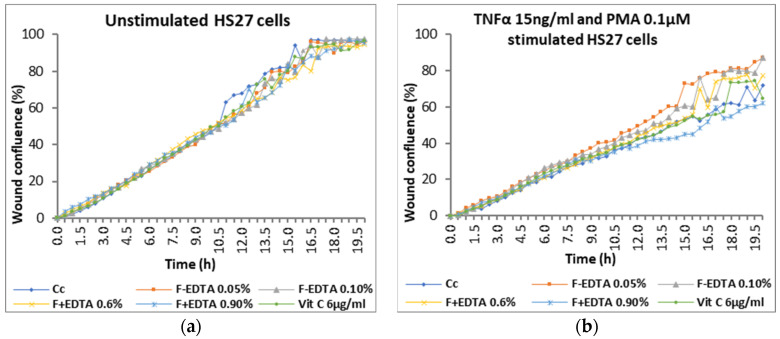
Evolution curves of wound confluence in 20 h under the influence of fish scale gelatin nanofibers without and with EDTA (F-EDTA, F+EDTA). Cells treated under normal conditions (**a**) and pro-inflammatory stimulated (TNFα 15 ng/mL and PMA 0.1 μM) (**b**).

**Figure 9:**
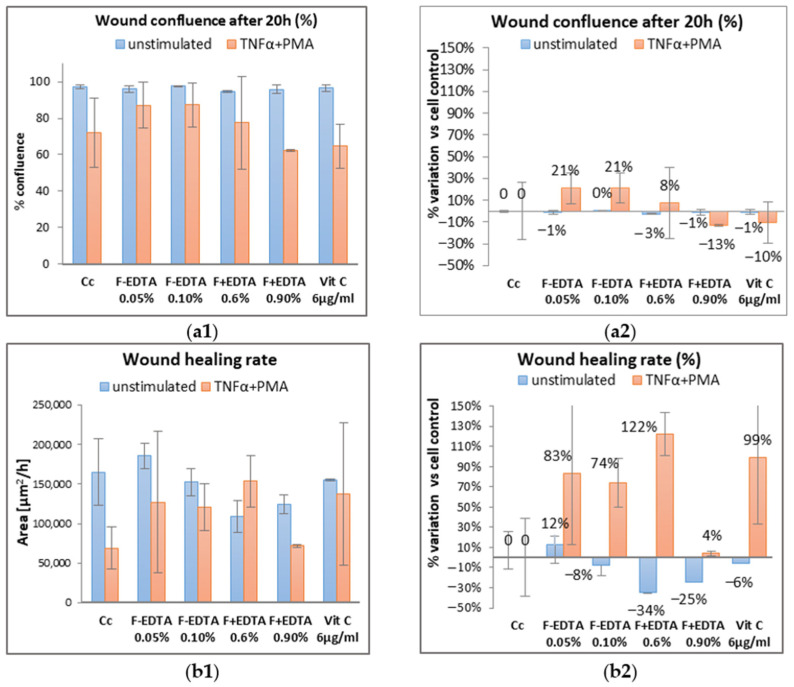
Evaluation of the wound healing process in the presence of the tested products, using image-based cellular analysis: Evolution of wound confluency (%) in 20 h (**a1**); wound healing rate—[µm^2^/h] (**b1**); calculation of the percentage variation compared to the cellular control (**a2**,**b2**). All numerical values are expressed as mean ± standard deviation (SD) (*p* > 0.05).

**Figure 10:**
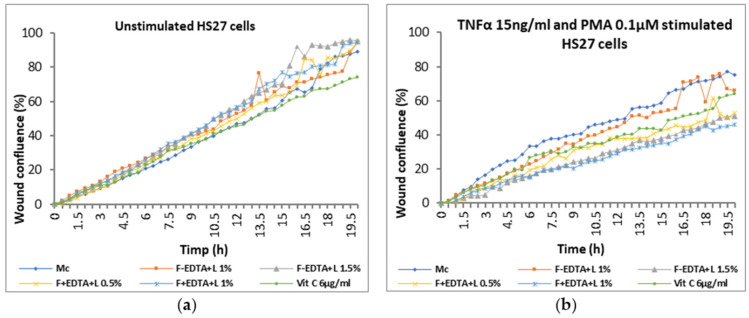
Evolution curves of wound confluence in 20 h under the influence of fish scale gelatin nanofibers without and with EDTA and lavender essential oil emulsion (F-EDTA+L and F+EDTA+L): cells treated under normal conditions (**a**) and pro-inflammatory stimulated (TNFα 15 ng/mL and PMA 0.1 μM) (**b**).

**Figure 11:**
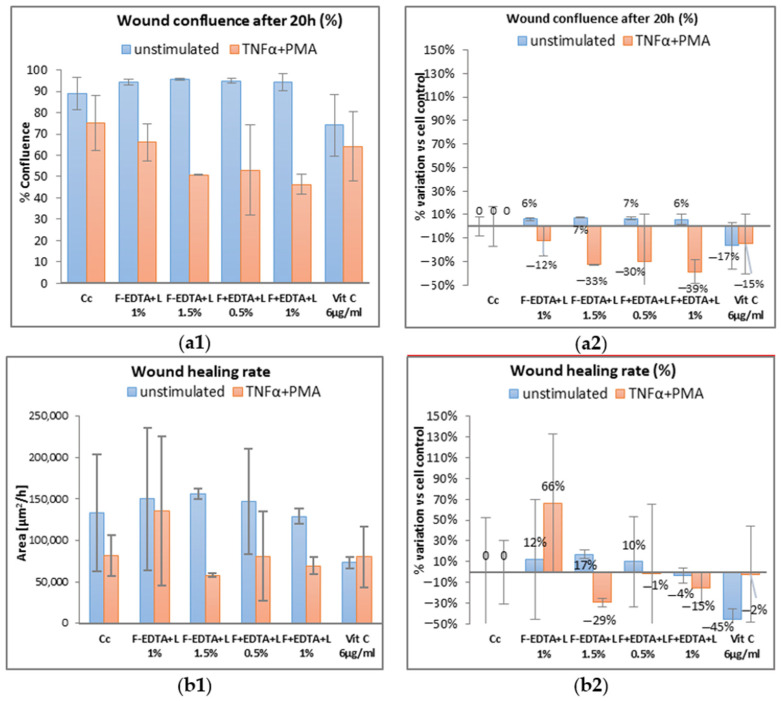
Evaluation of the wound healing process in the presence of the tested products containing lavender emulsion using image-based cellular analysis: Evolution of wound confluence (%) in 20 h (**a1**); wound healing rate—[µm^2^/h] (**b1**); calculation of the percentage variation compared to the cellular control (**a2**,**b2**). All numerical values are expressed as mean ± standard deviation (SD) (*p* > 0.05).

**Figure 12:**
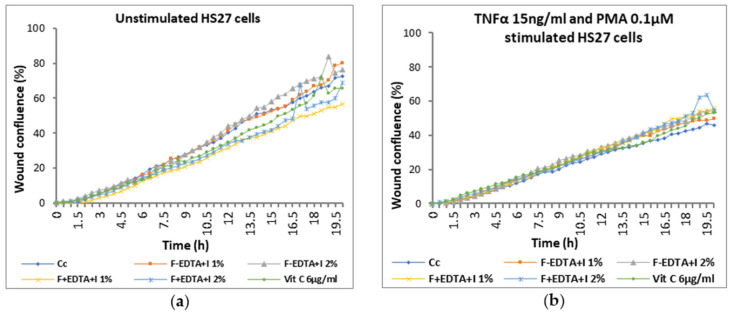
Evolution curves of wound confluence in 20 h under the influence of fish scale gelatin nanofibers without and with EDTA and essential oil emulsions (F-EDTA+I and F+EDTA+I): cells treated under normal conditions (**a**) and pro-inflammatory stimulated (TNFα 15 ng/mL and PMA 0.1 μM) (**b**).

**Figure 13:**
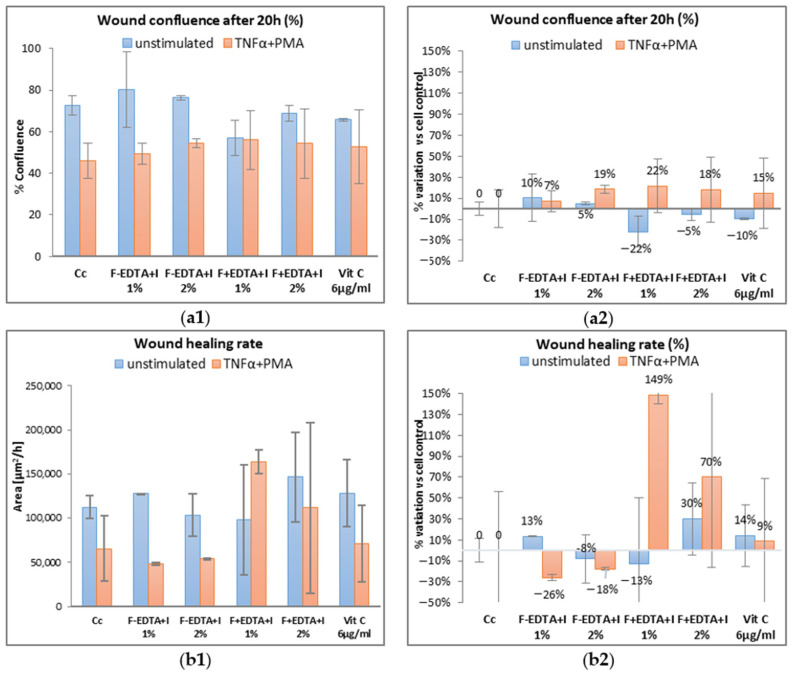
Evaluation of the wound healing process in the presence of the tested products containing immortelles emulsion using image-based cellular analysis: Evolution of wound confluency (%) in 20 h (**a1**); wound healing rate—[µm^2^/h] (**b1**); calculation of the percentage variation compared to the cellular control (**a2**,**b2**). All numerical values are expressed as mean ± standard deviation (SD) (*p* > 0.05).

Average kinetic wound confluence graphs are represented as average ± SD plotted for each sample concentration at every captured time point (Figure 8, Figure 9, Figure 10, Figure 11, Figure 12 and Figure 13).

Following the analysis of the data obtained in the wound healing test, at the level of fibroblasts treated under normal conditions (without stimulation), the percentage of wound confluence is over 90%, both in the case of the cell control and in the case of the cells treated with the samples of interest. On the other hand, in the case of nonspecific inflammation by stimulating the cells with TNFα 15 ng/mL and PMA 0.1 μM, the confluence of the wound increases by approximately 20% ± 0.50% in the presence of 0.05% F-EDTA and 0.1% F-EDTA. Regarding the wound healing rate, in the pro-inflammatory stimulated cells, there is an increase of 83% ± 0.358% and 74% ± 0.007%, respectively, in the cells treated with 0.05% F-EDTA, and 122% ± 0.41% in the presence of 0.6% F+EDTA (Figure 8 and Figure 9). For unstimulated cells, only 0.05% F-EDTA influences the wound healing process in a positive sense; the wound healing rate has a negative value for 0.1% F-EDTA.

After 20 h of treatment, in cells without stimulation, wound confluence is not significantly influenced, and the wound healing rate increases by up to 17% ± 0.77% in the case of treatment with 1.5% F-EDTA+L and 12% ± 1.43% in the presence of 1% F-EDTA+L (Figure 11b2). In the case of pro-inflammatory stimulated cells, the wound confluence decreases significantly compared to the cell control. The presence of 1% F-EDTA+L in the cellular environment induces a significant increase in the wound healing rate by up to 66% ± 0.25% (Figure 11b2).

In the case of nonspecific inflammation conditions by stimulating the cells with TNFα 15 ng/mL and PMA 0.1 μM, the confluence of the wound increases by approximately 19% ± 0.26% and 22% ± 0.089% in the presence of 2% F-EDTA+I and, respectively, 1% F+EDTA+I (Figure 13a2). Regarding the wound healing rate, for unstimulated cells, only 1% F-EDTA+I and 2% F+EDTA+I have a positive effect by increasing the percentage of wound healing with 13% ± 0.12% and 30% ± 0.30%, respectively (Figure 13b2).

In the case of nonspecific inflammation conditions by stimulating the cells with TNFα 15 ng/mL and PMA 0.1 μM, the confluence of the wound increases by approximately 19% ± 0.26% and 22% ± 0.089% in the presence of 2% F-EDTA+I and, respectively, 1% F+EDTA+I (Figure 13a2). Regarding the wound healing rate, for unstimulated cells, only 1% F-EDTA+I and 2% F+EDTA+I have a positive effect by increasing the percentage of wound healing with 13% ± 0.12% and 30% ± 0.30%, respectively (Figure 13b2). In the pro-inflammatory stimulated cells, which were treated with both concentrations of F+EDTA+I, there is an increase of 149% ± 1.41% (for 1% F+EDTA+I), respectively, 70% ± 2.87% (for 2% F+EDTA+I) of the wound healing rate (Figure 13b2). Similar rates of healing of 79% within 24 h were recorded by other authors [72].

Among the products with a potential contribution to the wound healing process by maintaining the homeostasis of the dermal extracellular matrix and restoring it and by a significantly increased wound healing rate, both for cells cultured under normal growth conditions, as well as for cells cultured by mimicking pro-inflammatory conditions, the following stand out: 0.05% F-EDTA; 1% F-EDTA+L; 2% F+EDTA+I nanofibers.

The wound-healing process is not only complex but also fragile, susceptible to delayed or stopped healing, leading to the formation of chronic nonhealing wounds. Wound healing is classically divided into two major phases (early phase and cellular phase), which are divided into several processes, namely, hemostasis, inflammation, proliferation, and remodeling [73]. Mimicking these in vitro inflammatory conditions was achieved by introducing pro-inflammatory agents (TNFα) and pro-oxidants (PMA) into the cellular system. Thus, the increased values of the wound healing rate, obtained as a result of the action of fish scale gelatin nanofibers, show their potential regenerating effect, as well as modulation of inflammatory processes specific to the dermal regeneration/restructuring process.

Rapid wound-healing activity performed by the induction of an artificial scratch on a confluent cell monolayer of nanofibers containing gelatin has been reported in other studies. In vivo wound healing measurement assessed for a bilayer scaffold prepared by electrospinning based on pork gelatin and fish collagen prepared at a ratio of 9:1, chitosan, and Lithospermi radix (LR) extract showed a recovery rate of 34.42 ± 5.9% for male rats, after forty days of treatment [74]. In another paper, an in vivo assessment was conducted on an electrospun scaffold composed of fish skin gelatin, polycaprolactone (PCL), and chitosan (CS). This scaffold was cross-linked with glutaraldehyde vapor and was evaluated as a skin substitute in a rat wound model where the complete skin removal had taken place [75]. After four weeks, the wound site exhibited only a 5.1 ± 2.3% healing compared to the control, possibly due to the rapid degradation of gelatin. Fish scale gelatin was also used for covering the phosphate-based glass fibers (PGFs) and investigated for cell migration evaluation by the wound scratching test [76]. The findings showed an increased percentage of wound closures, ranging from 69.8% to 70.9%, compared to the control and uncoated scaffold, which exhibited closure rates of 42.4% and 40.8%, respectively, following 24 h.

## 4. Conclusions

*Helichrysum italicum* and Lavandula latifolia essential oils were used for antimicrobial and wound healing enhancement of fish scale gelatin nanofibers prepared by coaxial electrospinning. Fish scale gelatins with good spinnable properties were extracted with and without EDTA treatment and with high yields of 25.06% ± 0.40% and 33.41% ± 0.32%, respectively. The antimicrobial properties of fish scale gelatin nanofibers with *Helichrysum italicum* and Lavandula latifolia essential oils were substantially improved as compared to fish scale nanofibers without essential oils recommending the new products for topical and pharmaceutical products. The noncytotoxicity range of concentrations of nanofibers with and without Helichrysum italicum and Lavandula latifolia essential oil emulsions was found to be 0.05–5%. The presence of essential oils tended to improve the noncytotoxicity concentrations of fish scale nanofibers. The “scratch-test”, which involved the creation of an artificial wound in the cell monolayer, reached confluence, and the continuous monitoring of cell migration on its surface for 20 h in the presence of test substances and stimuli showed that the most effective wound healing rate was 149% under the influence of fish scale gelatin nanofibers with EDTA and *Helichrysum italicum* essential oil emulsion.

In this study, we explored the in vitro wound-healing potential of the fish scale gelatin nanofibers with and without essential oils, focusing on their physicochemical properties, biocompatibility, antimicrobial activity, and wound-healing efficacy. Biocompatibility assessment of the active components revealed noncytotoxic behavior in specific concentration ranges in the viability assay using human fibroblast (HS27) cells. The wound-healing assay results indicated that fish scale gelatin nanofibers showed promising wound closure after 20 h of application, highlighting its regenerative potential on fibroblasts treated both under normal conditions and pro-inflammatory stimulated.

## 5. Patent

Gaidau, C.; Rapa, M.; Predescu, C.; Stanca, M.; Alexe, C.A. Nanofibers from fish scale collagen and process to obtaining therefore, OSIM A00782 from 29.11.2022, published in 2023, https://www.osim.ro/images/Publicatii/Inventii/2023/inv_03_2023.pdf, page 23 (accessed on 10 April 2023).

## Figures and Tables

**Table 1:** The fish scale gelatin nanofibers without and with the lavender and immortelle essential oil emulsions prepared by electrospinning.

Sample Abbreviation	Description
F-EDTA	Nanofibers of fish scale gelatin without EDTA
F+EDTA	Nanofibers of fish scale gelatin with EDTA
F-EDTA+L	Nanofibers of fish scale gelatin without EDTA, with lavender essential oil emulsion
F-EDTA+I	Nanofibers of fish scale gelatin without EDTA, with immortelle essential oil emulsion
F+EDTA+L	Nanofibers of fish scale gelatin with EDTA and lavender essential oil emulsion
F+EDTA+I	Nanofibers of fish scale gelatin with EDTA and immortelle essential oil emulsion

**Table 2:** Electrospinning parameters used for the preparation of fish scale gelatin nanofibers.

Parameters	F-EDTA	F+EDTA	F-EDTA+L	F-EDTA+I	F+EDTA+L	F+EDTA+I
Flow rate (mL/h)	1.4	0.8	0.9/0.3	1.2/0.7	1.9/0.7	0.7/0.3
Voltage supply (kV)	22.15	22.11	23.60	23.60	23.60	23.60
Collector distance (mm)	120	120	120	120	120	120

**Table 3:** Physical–chemical characteristics of fish scale gelatins.

Characteristics	Gelatins Extracted from Fish Scales	
	F-EDTA	F+EDTA
Dry substance, %	3.61 ± 0.35	4.82 ± 0.35
Total ash, %	0.02 ± 0.01	nd
pH (1:10), pH units	6.59 ± 0.10	4.91 ± 0.10
Bloom test, g	249.40	362.10
Relaxation, %	34.50	14.30
Viscosity, CP	3.35 ± 0.05	4.00 ± 0.08
Conductivity, μS/cm	344 ± 0.10	442 ± 0.10
MW, mol/g	292,008	346,816
Polydispersity	1.01	1.03

nd: not detected.

**Table 4:** Molecular weight compositions for F-EDTA and F+EDTA.

Band No.	F-EDTA		F+EDTA	
	MW (kDa)	Band (%)	MW (kDa)	Band (%)
1	250	4.4	250	8.1
2	250	3.1	249.8	30.4
3	238.9	2.7	160.3	3.5
4	181.9	5.9	147.1	3.1
5	147.3	9.9	129.7	19.9
6	134.6	15.8	119.5	10.9
7	119.7	6.9	107.3	3.2
8	105.6	1.8	96.0	4.0
9	79.4	20.3	88.1	2.7
10	69.9	5.7		
11	60.4	0.7		
12	55.8	2.3		
13	44.7	5.6		
14	38.3	3.2		
15	25.7	3.2		
16	20.4	2.2		
17	15	0.7		
18	10	1.7		
19	10	1		
20	10	2.3		

**Table 5:** Elemental composition of fish scale gelatin nanofibers.

Nanofibers	C		N		O		P		Ca	
	Wt%	At%	Wt%	At%	Wt%	At%	Wt%	At%	Wt%	At%
F-EDTA	45.03	58.87	24.49	27.45	11.48	11.27	1.15	0.58	1.03	0.40
F+EDTA	46.98	62.76	22.63	25.92	9.25	9.28	0.27	0.14	0.15	0.06

**Table 6:** Antimicrobial activity of *Helichrysum italicum* and Lavandula latifolia essential oils.

Essential Oils/ Reference	Inhibition Zones, mm		
	*E. coli*	*S. aureus*	*C. albicans*
*Helichrysum italicum*	9.83 ± 0.76	31.50 ± 0.50	11.67 ± 0.58
*Lavandula latifolia*	13.50 ± 1.50	46.50 ± 0.50	17.33 ± 3.51
Ampicillin	8.00 ± 2.00	32.67 ± 2.52	21.32 ± 0.68

**Table 7:** MIC values of *Helichrysum italicum* and Lavandula latifolia essential oils.

Microorganisms	MIC, µL × mL^−1^		
	*Helichrysum italicum*	*Lavandula latifolia*	Gentamicin
*E. coli*	1.56 ± 0.00	6.25 ± 0.00	0.70 ± 0.00
*S. aureus*	0.63 ± 0.00	2.60 ± 0.90	0.80 ± 0.00

**Table 8:** Microbial loads of fish scale gelatin nanofibers without and with essential oil emulsions.

Fish Scale Gelatin Nanofibers and Essential Oils Emulsions	TAMC, CFU/g	TYMC, CFU/g
F-EDTA	5500.00 ± 4.36	45.00 ± 3.00
F-EDTA+L	22.33 ± 9.29	14.66 ± 1.16
F-EDTA+I	31.33 ± 7.76	20.33 ± 1.53
F+EDTA	2300.00 ± 2.00	22.00 ± 1.73
F+EDTA+L	14.00 ± 2.00	8.33 ± 2.08
F+EDTA+I	21.33 ± 0.58	11.33 ± 2.52
*Lavandula latifolia* essential oil emulsion (L)	0	0
*Helichrysum italicum* essential oil emulsion (I)	0	0

**Table 9:** Identification of *S. aureus*, *E. coli*, and *C. albicans* on fish scale nanofibers and essential oils emulsions.

Fish scale Gelatin Nanofibers and Essential Oils Emulsions	*Staphylococcus aureus*	*Escherichia coli*	*Candida albicans*
F-EDTA	Positive	Positive	Negative
F- EDTA+L	Negative	Negative	Negative
F-EDTA+I	Negative	Negative	Negative
F+EDTA	Negative	Positive	Negative
F+EDTA+L	Negative	Negative	Negative
F+EDTA+I	Negative	Negative	Negative
*Lavandula latifolia* essential oil emulsion (L)	Negative	Negative	Negative
*Helichrysum italicum* essential oil emulsion (I)	Negative	Negative	Negative

**Table 10:** Maximum toxicity dose of the fish scale gelatin nanofibers containing essential oil emulsions tested on the HS27 cell line.

Sample	Maximum Toxicity Dose, % (±SD)
F-EDTA	1 ± 0.008
F+EDTA	1 ± 0.007
F-EDTA+L	2 ± 0.018
F+EDTA+L	1.5 ± 0.050
F-EDTA+I	4 ± 0.037
F+EDTA+I	4 ± 0.051

## Data Availability

The data presented in this study are available on request from the corresponding author.

## Supplementary Materials

The following supporting information can be downloaded at: https://www.mdpi.com/article/10.3390/pharmaceutics15122692/s1, Figure S1: Chemical structure for: (**a**) Neryl acetate; (**b**) Linalool; Figure S2: Emulsions of lavender and immortelle essential oils; Figure S3: Electrospinning of fish scale gelatin dispersions on polypropylene sheet; Figure S4: GP chromatograms for: (**a**) F-EDTA, and (**b**) F+EDTA; Figure S5: SDS–PAGE electrophoresis gels band analyses and the molecular weight distribution of fish scale gelatins. (**a**) F-EDTA; (**b**) F+EDTA.
