# Supplementary material for: Fish Scale Gelatin Nanofibers with Helichrysum italicum and Lavandula latifolia Essential Oils for Bioactive Wound-Healing Dressings
Source: Pharmaceutics. 2023 Nov 28;15(12):2692. doi: 10.3390/pharmaceutics15122692 (PMC10747005; doi:10.3390/pharmaceutics15122692)
Supplement: Supplementary file 1 [file pharmaceutics-15-02692-s001.zip › pharmaceutics-2667190-supplementary.pdf]

pharmaceutics

Supplementary Material

Fish Scale Gelatin Nanofibers with *Helichrysum Italicum and Lavandula Latifolia Essential Oils* for Bioactive Wound-Healing Dressings

Carmen Gaidau [1], Maria Râpă [2,*], Maria Stanca [1,*], Mariana-Luiza Tanase [3], Laura Olariu [3], Rodica Roxana Constantinescu [1], Andrada Lazea-Stoyanova [4], Cosmin-Andrei Alexe [1] and Madalina Tudorache [5]

1 The National Research & Development Institute for Textiles and Leather-Division Leather and Footwear Research Institute, Bucharest 31251, Romania; carmen.gaidau@icpi.ro (C.G.); rodica.roxana@yahoo.com (R.R.C.); cosminandrei.alexe@icpi.ro (C.-A.A.)
2 Faculty of Materials Science and Engineering, National University of Science and Technology Politehnica Bucharest, Bucharest 060042, Romania; maria.rapa@upb.ro
3 SC Biotehnos SA, 3–5 Gorunului Street, Otopeni 075100, Romania; luiza.craciun@biotehnos.com (M.-L.T.); lolariu@biotehnos.com (L.O.)
4 National Institute for Laser, Plasma and Radiation Physics, 409 Atomistilor Street, Magurele, Ilfov 077125, Romania; andrada@infim.ro
5 Laboratory for Quality Control and Process Monitoring, Faculty of Chemistry, University of Bucharest, 4-12 Regina Elisabeta Boulevard, Bucharest 030018, Romania; madalina.sandulescu@g.unibuc.ro
* Correspondence: maria.rapa@upb.ro (M.R.); maria.stanca@icpi.ro (M.S.)

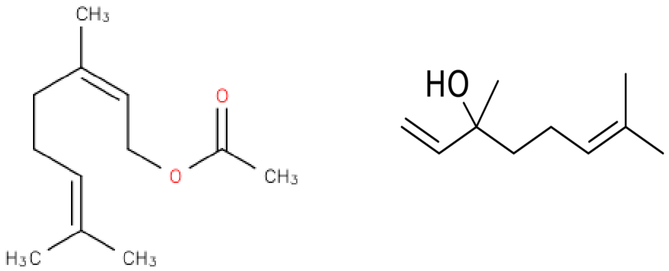

(a) **(b)**

Figure S1. Chemical structure for: (a) Neryl acetate; (b) Linalool.

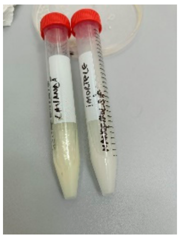

Figure S2. Emulsions of lavender and immortelle essential oils.

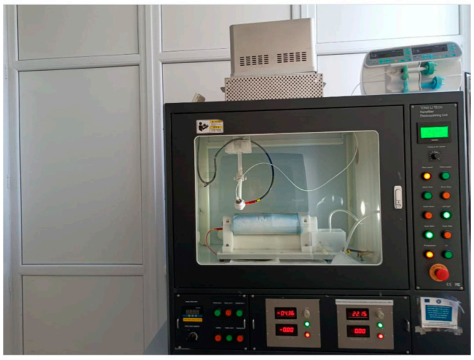

Figure S3. Electrospinnig of fish scale gelatin dispersions on polypropylene sheet.

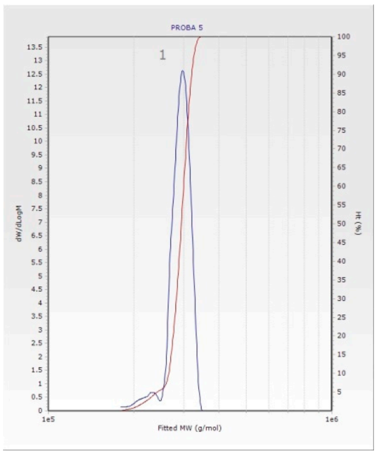

(a)

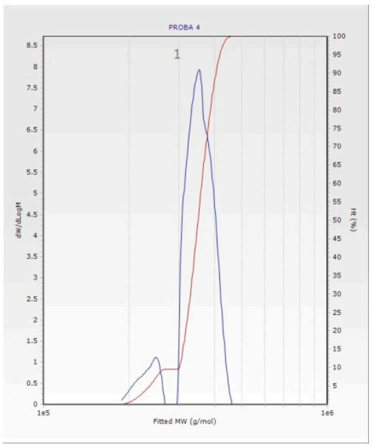

(b)

Figure S4. GP chromatograms for: (a) F-EDTA, and (b) F+EDTA.

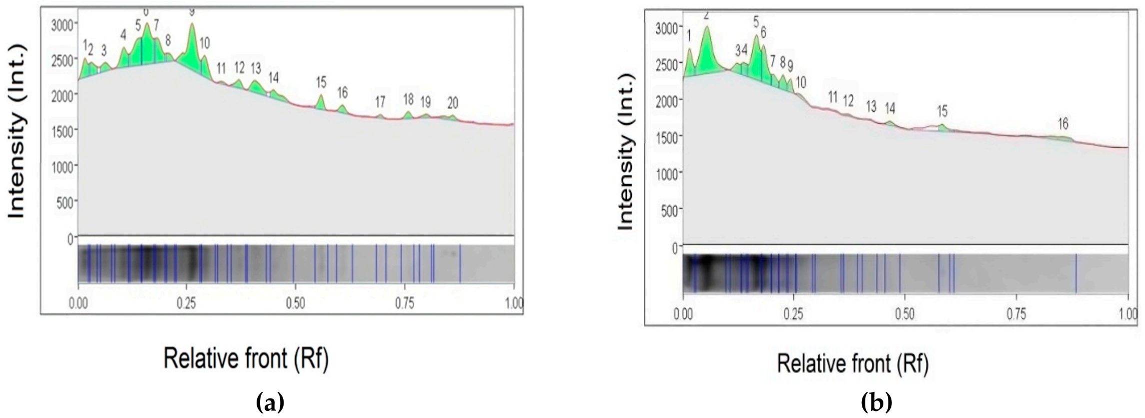

Figure S5. SDS-PAGE electrophoresis gels band analyses and the molecular weight distribution of fish scale gelatins. (a) F-EDTA; (b) F+EDTA**.**
